# Miss to the Right: The Effect of Attentional Asymmetries on Goal-Kicking

**DOI:** 10.1371/journal.pone.0012363

**Published:** 2010-08-24

**Authors:** Michael E. R. Nicholls, Tobias Loetscher, Maxwell Rademacher

**Affiliations:** 1 School of Psychology, Flinders University, Bedford Park, South Australia, Australia; 2 Department of Psychological Sciences, University of Melbourne, Parkville, Victoria, Australia; CNRS, France

## Abstract

Cerebral asymmetries for spatial attention generate a bias of attention – causing lines to be bisected to the left or right in near (within reach) and far (outside reach) space, respectively. This study explored whether the rightward deviation for bisecting lines in far space extends to tasks where a ball is aimed between two goal-posts. Kicking was assessed in a laboratory and a real-life setting. In the laboratory setting, 212 participants carried out three conditions: (a) kick a soccer ball at a single goal post, (b) kick a soccer ball between two goal posts and (c) use a stick to indicate the middle between two goal posts. The goals were placed at a distance of 4.0 m. There was no deviation in the one-goal kicking condition – demonstrating that no asymmetries exist in the perceptual motor system when aiming at a single point. When kicking or pointing at the middle between two goal posts, rightward deviations were observed. In the real-world setting, the number of misses to the left or right of goal (behinds) in the Australian Rules football for the 2005–2009 seasons was assessed. The data showed more rightward deviations for kicks at goal. Combined, the studies suggest that the rightward deviation for lines placed in far space extends to the kicking of a football in laboratory and real-life settings. This asymmetry in kicking builds on a body of research showing that attentional asymmetries impact everyday activities.

## Introduction

The apparent left/right symmetry of the human body at a surface level belies a number of significant functional brain asymmetries that affect perception and action. Asymmetries in attention are most clearly evident in patients with clinical neglect. Following damage to the right parietal damage, neglect patients show an inability to perceive and respond to stimuli located in the contralesional (left) hemispace [1]. As a result, the patient may collide with objects located on the left when walking [2] or operating a wheelchair [3], [4]. The rightward bias in attention is clearly evidenced in clinical tests, such as line bisection. When asked to mark the middle of a horizontal line, neglect patients place the bisection far to the right of true centre [1], [5].

While neglect patients show a strong attentional asymmetry, the general population shows a subtle attentional bias. When asked to bisect a line placed in near space, intact-brain participants reliably place the bisector slightly to the left of true centre [6]. This bias operates in the opposite direction to clinical neglect, and for this reason, it is often called ‘pseudoneglect’ [7]. The link between clinical neglect and pseudoneglect seems apt given that they both originate from the same neural mechanisms located in the right posterior parietal cortex [8], [9], [10]. Furthermore, they are affected in an analogous fashion by changes in stimulus length [11], prismatic adaptation [12], [13] and attentional cueing [14]. Pseudoneglect may be the product of right hemisphere specialisation for spatial attention and the stronger connections between this hemisphere and the contralateral hemispace [15].

Attentional asymmetries change as a function of near and far space. Work with primates [16] and human populations [17] show that distinct neural circuits are used for interacting with objects in near (within reach) and far (outside reach) space. A dissociation in the neural mechanisms that underlie attention in near and far space has also been observed in the general population. Bjoertomt, Cowey and Walsh [8] found that transcranial magnetic stimulation over the right posterior parietal cortex affected line bisection in near space whereas stimulation over the right ventral occipital lobe affected attention in far space.

Besides having different neural substrates, near and far space also affects the distribution of attention between the left and right hemispaces. Longo and Lourenco [19] asked participants to bisect lines placed at distances between 300 mm and 1.2 m from the participant using a laser pointer. A leftward bisection bias was observed for lines that were near (within reach), which reversed to a rightward bisection bias for lines placed further away. To investigate the effect of tool use, Longo and Lourenco [18] also asked participants to make the bisections using a stick. In this case, a leftward bisection bias was observed irrespective of viewing distance. Studies with primates [19] and humans [20] reveal that tool use can bring distant object within ‘reach’ and activate brain centres that are normally associated with objects in peripersonal space.

Asymmetries in attention have the potential to affect subtly many of our interactions with the every-day environment. Turnbull and McGeorge [21] asked undergraduates if they had collided with anything and on which side the collision occurred. Although a bias towards more rightward collisions failed to reach significance, they did find an association between asymmetries in line bisection and asymmetries in collisions. Thus, people who bisected lines to the right also had more rightward collisions (and vice-versa for leftward bisectors). A bias towards rightward collisions has been observed under laboratory conditions when participants walk through a narrow doorway [22], [23]. This asymmetry in collisions is not limited to ambulatory tasks and has also been found for the operation of electric wheelchairs [24].

Besides affecting everyday activities, asymmetries in attention may also influence sporting performance where left/right spatial judgements are often crucial. Roberts and Turnbull [26] asked novices to putt golf-balls towards a cup and found more misses to the right. They also found an association between the asymmetry for putting and asymmetries for line bisection. Roberts and Turnbull therefore suggested that the putting error was related to attentional asymmetries of the type described by Longo and Lourenco [18] for stimuli placed in far space.

In the study by Roberts and Turnbull [25], it is not entirely clear whether the putting task actually involved a bisection judgement. That is, participants aimed towards a cup, but did not have to bisect a segment of space. A better understanding of how attentional asymmetries affect performance might come from sports where a ball is aimed between two goal posts (e.g. rugby, Australian rules football, gridiron, hockey and soccer). With this in mind, the present study investigated asymmetries in kicking a football (soccer) between two goal posts.

Participants completed three conditions. In the *one-goal kicking* condition, kicks were aimed at a single goal post placed at a distance of 4.0 m. This condition provides a baseline measure of kicking ability. If a deviation to the left or right is observed in this condition, it would indicate a mechanical/motor asymmetry perhaps related to the foot that is used, or the curve that is generated. In the *two-goal kicking* condition, participants aimed their kicks between two goal posts. Because this condition measures bisection biases in far space, it was expected that participants would bisect the space slightly to the right of true centre and therefore kicks would deviate to the right. In the *pointing* condition, participants used a long stick to indicate the middle between the two goal posts. Previous research has indicated that far objects can be brought into near space through tool use when they are placed at a distance of 1.2 m [18] (see above). Because the distance in the current study (4.0 m) is so far in excess of previous studies, it was expected that the goal posts would remain in far space. Rightward bisections were therefore predicted. For both the pointing and two-goal kicking conditions, the distance between the goal posts was varied between 500 mm and 1.2 m. In line with a large body of research investigating the effect of line length of bisection asymmetries [see 26], it was expected that the bisection biases would increase for wider goals. Roberts and Turnbull [25] observed an association between line bisection and deviation in putting. While an association between different measures of attentional asymmetry is sometimes observed [23], a number of studies have found no effect [24], [27]. The association between the two-goal kicking and pointing data were examined with a correlational analysis.

**Figure 1:**
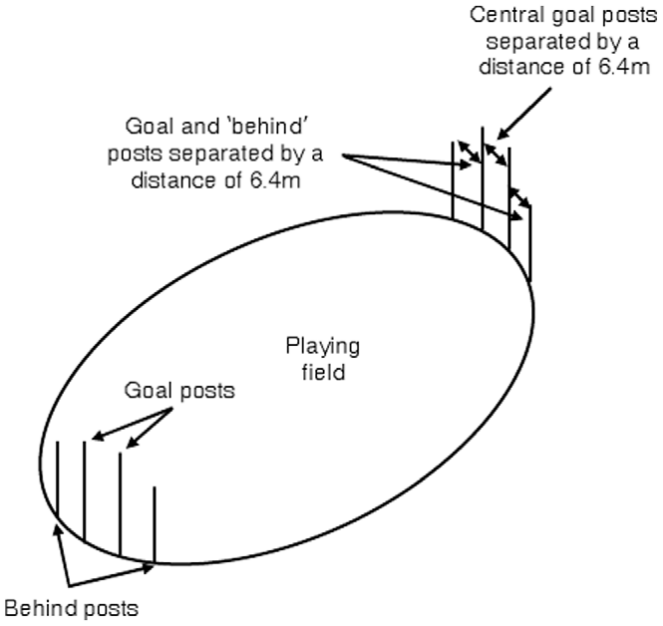
Illustration of a typical Australian Rules Football ground, showing the arrangement of the ground and the goal and behind posts. If the ball is kicked between the goal posts, a goal is scored. If the ball misses to the left or right, but is within one of the behind posts, a behind is scored. The field can be between 135–185 m long and 110–155 m wide.

In addition to examining kicking asymmetries in the laboratory, we explored kicking asymmetries in the real ‘sporting’ world. Australian Rules football is a game where the objective is to kick a football between two goal posts (worth six points). If the kick misses to either side of the goal, but passes within one of the ‘behind’ posts, a ‘behind’ is scored (worth one point). Figure 1 shows the layout of an Australian Rules football ground and the goal and behind posts. The kick can be taken at any distance from the goal posts, but kicks longer than 60 m are rare. Most kicks are taken by dropping the ball from the hands and kicking. Kicks at goal are sometimes taken on the run, and on other times, are taken as a free kick is the ball is ‘marked’. Unlike sports like soccer or hockey, there is no goalie who plays a significant role in deflecting shots at goal and there is no limit to the height of the goal. Asymmetries related to the defensive players are therefore unlikely to affect whether players miss to the left or right. By examining the number of behinds scored to the left and right, Australian Rules football should provide a useful means of testing asymmetries in kicking on the sporting field. Data were collected from the 2005–2009 Australian Football League (AFL) seasons. In each season 185 games are played between 16 teams. We were able to gain access to information related to whether a left or right behind was scored and the angle and distance at which the kick was taken. While behinds can be scored if the ball hits one of the goal posts, or if a player touches the ball with their hand, the present analysis was limited to behinds scored by a kick that missed to the left or right of the goal. It was predicted that more behinds to the right (from the kicker's perspective) would be scored.

## Methods

### Ethics statement

This study was conducted according to the principles expressed in the Declaration of Helsinki and had approval from the Melbourne University Human Research Ethics Committee.

All participants provided written informed consent for the collection of data and subsequent analysis.

### Participants

Undergraduate students (n = 245) participated as part of their course requirement. To provide a homogeneous sample, only individuals who kicked the ball with their right foot and who were right-handed [28] were included in the data analysis. This left 212 participants (m = 50, f = 162) with an average right foot preference of +2.51 [30] and a modal age of 19 years. The large majority of participants were novices in relation to playing soccer.

### Apparatus & Stimuli

A soccer ball was used for the kicking trials and a 4.0 m length of 20 mm plastic tube was used for the pointing trials. The goal posts were made of 1.0 m lengths of 20 mm white plastic tube. The posts were fixed in a vertical position along the top of the sensor array and were designed so that the width of the goals could be adjusted between participants. The sensor array consisted of a horizontal series of 28 micro-switches (Omron, ‘V3’ style, long lever). Each switch was separated by a horizontal distance of 70 mm. The separation was chosen so that contact between the ball and the array always activated at least one switch. The sensor array was 200 mm high and 2.0 m long and the face was covered in black cloth to hide the switches from the participant. The switch array was connected by a 6.0 m lead to a response array containing 28 LEDs. Activation of any the 28 switches resulted in the illumination of a corresponding LED along the response array. The LEDs were arranged in a line and coded from −14 for a kick to the far left or the sensor array to +14 for a kick to the far right of the array. The response array was latched so that the LEDs remained illuminated until the experimenter hit a reset button. To measure the accuracy of pointing trials, a ruler was fixed to the back of the sensory array.

### Procedure

Participants performed bisections from a horizontal line placed 4.0 m in front of the goal or goal posts. There were three conditions. In the *one-goal kicking* condition, one goal post was placed in the centre of sensor field and participants aimed their kicks at the goal. In the *two-goal kicking* condition, two goal posts were placed around the centre of the array, separated by a horizontal distance of 500 mm, 900 mm and 1500 mm. The different widths were varied between participants at random. Participants were asked to aim their kicks at the middle of the goal posts. Accuracy, and not speed, was encouraged. For both of the kicking conditions, the accuracy of the kick was read from the response array. If a single LED was illuminated, such as +2, the response was recorded as +2. If two or three LEDs were activated, the mean was taken. For example, if −1 and +1 were activated, a score of 0 was recorded. The ball never activated more than 3 LEDs. This procedure allowed the position of the kick to be calculated with an accuracy of ±35 mm. Participants were asked to kick the ball with the foot they normally used. The same foot was used for all trials.

**Figure 2:**
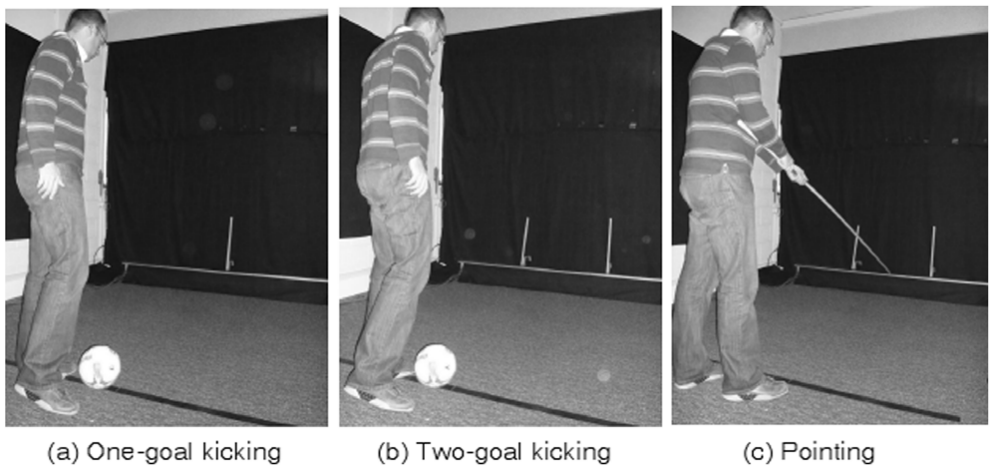
Illustration of the set-up for the: (a) One-goal kicking, (b) two-goal kicking and (c) pointing conditions.

In the *pointing* condition, participants held a 4.0 m length of plastic tube with both hands. The far end of the pointer lay on top of the sensor array. The left/right starting position of the pointer was varied between trials. Participants were asked to slide the pointer along the top of the sensor array until it lay between the two goal posts. The positioning of the goal posts was the same as the two-goal kicking condition. When the participant indicated that they were satisfied with their bisection, the experimenter moved to the back of the array and read the accuracy of the bisection from the ruler. See figure 2 for an illustration of the three conditions.

For each of the three conditions, participants performed two kicks/points (i.e participants completed six trials in all). The order in which the three conditions were administered was balanced. The experimenter also administered a handedness questionnaire [28] and a footedness questionnaire [29].

## Results

### Experimental data

**Figure 3:**
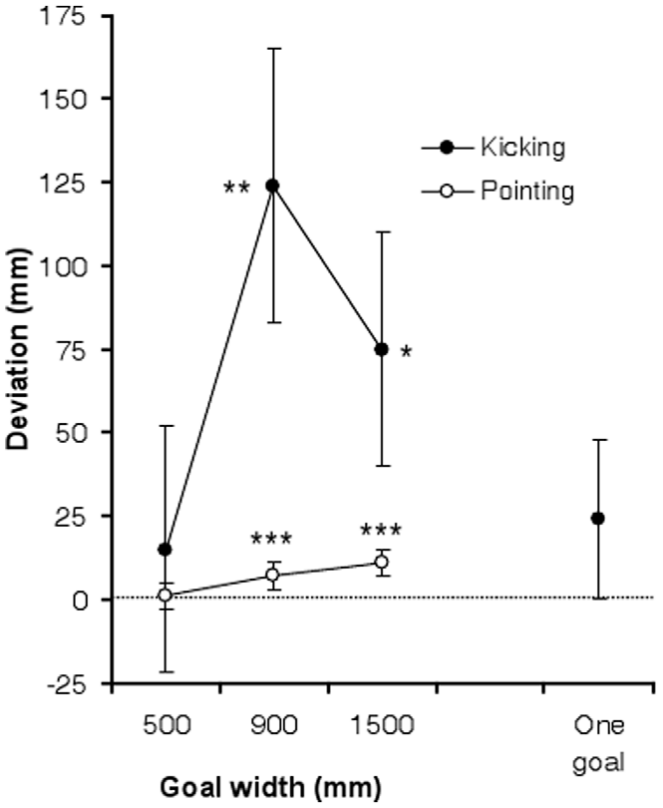
Mean deviations in mms for the two-goal pointing and kicking conditions across the different goal widths (with ±SE bars). Negative and positive reflect a deviation to the left and right, respectively. The results of t-tests comparing the conditions with zero are shown (* p<.05, **p<.005, ***p<.001). The mean for the one-goal kicking condition is shown in the far right of the figure.

The kicking data collected from the response array were converted into measurements of mms by scaling them to the actual size of the sensor array. The mean deviation for the one-goal kicking condition was +23.9 mm (SD = 335.6). A one-sample t-test comparing the means with zero revealed that the distribution of means was not biased to either the left or right [t(211) = 1.04, ns]. The mean deviation for the two-goal kicking condition was +68.2 mm (SD = 318.4). A one sample t-test revealed that the distribution of means was significantly biased towards the right [t(211) = 3.12, p<.005]. To test the effect of goal-width on deviations in the two-goal kicking condition, an ANOVA was conducted with width (500, 900 & 1500 mm) as a between participants factor. While the ANOVA revealed no significant effect of width [F(2,209) = 1.90, ns], one-sample t-tests revealed that the rightward deviation was significant for widths of 900 and 1500 mm, but not for a width of 500 mm (see Figure 3).

Analysis of the pointing data revealed a mean bias of +6.3 mm (SD = 14.9), which was significantly to the right of zero [t(211) = 6.13, p<.001]. An ANOVA with width as a between participants factor revealed a significant effect of width [F(2,209) = 9.92, p<.001]. As shown in figure 3, the rightward deviation was significant for the two greater widths, but not for the smallest width. There was no significant correlation between the two-goal kicking and pointing conditions (*r*(212) = .08, ns). To control for the fact that the effect of goal width may be different for the two-goal kicking and pointing conditions, separate correlations were calculated between two-goal kicking and pointing within each of widths. None of the correlations approached statistical significance.

### Australian Rules Football data

**Figure 4:**
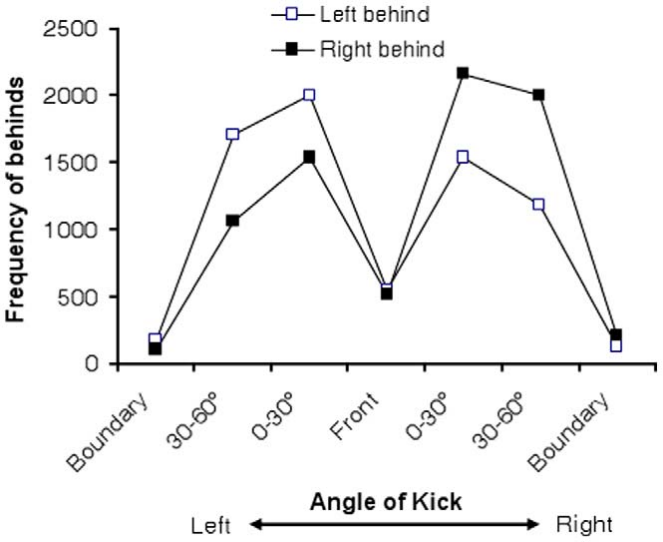
Frequency of left and right behinds during the 2005–2009 AFL seasons broken down by the angle and side at which the kick was taken.

In the five years that were sampled, there were 14,827 kicks, which resulted in a behind being scored. There were 7,260 behinds to the left and 7,567 behinds to the right. Note that, like the experimental study, left and right are scored from the kicker's perspective. A chi-square analysis revealed that right behinds were more likely to occur than left behinds (*x* ^2^ (1) = 6.35, p<.05). The data, broken down the angle at which the kick was taken, are shown in Figure 4. There graph shows that there were more right behinds than left behinds. In addition, there is a clear interaction between the side on which the kick was taken and the type of behind scored (*x* ^2^ (6) = 560.83, p<.001). Kicks from the right side were more likely to result in right behinds and vice-versa for kicks from the left side.

## Discussion

In the one-goal kicking condition, there was no significant deviation to the left or right. This condition was designed to provide a baseline measure of kicking ability where participants aimed their kick at a single point of space (a single goal post). In this regard, the one-goal kicking condition is similar to the task used by Roberts and Turnbull [25], which required participants to putt a golf ball toward a single point (a cup). It is therefore interesting that Roberts and Turnbull observed a rightward deviation, whereas we did not. Because both hands are used when putting, the rightward deviation cannot be the result of a simple handedness effect. When holding a putter, however, the right hand is usually held lower down the shaft than the left if the person is right-handed. It is therefore possible that the asymmetry observed by Roberts and Turnbull may be related to some bias in the swing, which causes the ball to deviate to the right. The explanation, however, does not account for the association between putting and line bisection observed by Roberts and Turnbull [25]. For the purposes of the present study, it can be concluded that participants do not show an asymmetry when kicking towards a single goal post and therefore there are no obvious mechanical/motor asymmetries that cause the ball to deviate.

The two-goal kicking condition measured biases in kicking when participants bisected the space between two goal posts. In this case, participants kicked the ball to the right of centre. The fact that a rightward deviation was observed for this condition, but not for the one-goal condition, suggests that the deviation is related to the act of bisecting the space between the goal posts. Longo and Lourenco [18] demonstrated that lines are bisected to the right of centre when they are placed in far space. Given that the goal posts were outside reach, and that participants were asked to aim for the centre of the goal, it seems reasonable to conclude that the rightward deviation observed in the current study has a similar origin to the one observed by Longo and Lourneco. The effect of goal-width supports this proposition. There is a large body of research showing that line bisection biases increase for longer lines in the general population [see 26]. The current study found no significant main effect of goal-width on deviation – most probably because of the high level of variability in kicking skill. Nevertheless, the rightward deviation was only significant for the wider goals.

The pointing condition also revealed a bias towards bisecting the space to the right of true centre. Research by Longo and Lourenco [18] demonstrated that objects placed in far space could be brought into near space if the bisection was made using a tool such as a stick. In the current study, a stick was also used to make the bisection. Therefore, like Longo and Lourenco, a leftward deviation might be expected. However, the distances used in the current study (4.0 m) is far in excess of the distance used by Longo and Lourenco (1.2 m). Although an effect of tool use on near and far space has been shown by a number of researchers for objects located within 1.2 m [18], [30] we know of no study that has used tools at greater distances. For these greater distances, we suspect that the difficulty of wielding a long stick, the flexibility of the stick and the distance itself all militate against a conversion of far into near space. The rightward deviation observed in the current study also showed an effect of goal-width whereby the rightward deviation increased for the wider goals. This effect is also in line with the line bisection literature [26].

The association between deviations in the pointing condition and the two-goal kicking condition was examined using a correlational analysis. It would seem reasonable to assume that, if the rightward deviations are the product of the same underlying cognitive/neurological mechanisms, there should be a relationship between them. Despite this, no correlation was found. While Roberts and Turnbull [25] found an association between putting and line bisection deviations, it was significant for only one out of four possible associations. Similarly, in a study examining associations between various measures of pseudoneglect, Luh [27] found that most yielded a leftward deviation – but there was no significant correlation between the measures. The association between different measures of attentional asymmetry therefore appear weak and this may be exacerbated by using different effectors (foot and hand). In support of this, the kicking condition was also associated with considerably more error than the pointing condition. In addition, while it may be reasonable to propose that differences in the direction of attentional asymmetry should be consistent within individuals, there may be less reason for assuming that the degree of asymmetry will be consistent across tasks.

The AFL kicking data produced an interesting set of results. Like the experimental data, when players were aiming for the middle between the two goal posts, they deviated towards the right and scored more rightward behinds. The same biases that led to rightward deviations for the pointing and two-goal kicking conditions may account for the rightward deviations in AFL. That is, when taking a kick at goal, players estimate the midpoint between the goal posts. Because of asymmetries between the cerebral hemispheres in the way spatial attention is allocated, the estimated midpoint is shifted to the right in far space. Players then aim for this point, and because of a normal distribution of error, misses are more likely to result in rightward behinds. It should be acknowledged, however, that Australian Rules Football is a highly dynamic game and players are virtually free to occupy any place on the ground. As a result, in addition to simply aiming for the centre between the two goal posts, the kicker is also trying to avoid other players. While we do not believe that there should be any asymmetry in the interaction between players which would account for the rightward kicking bias, we acknowledge that the interaction between players will reduce the accuracy of kicking. By sampling such a large number of kicks, we hope to observe an effect despite this ‘noise’. It should also be noted that we cannot rule out the possibility that the rightward deviation for the AFL data is related to the foot that was used to kick the ball. Unfortunately, the current set of data does not have information related to which foot was used to kick the ball.

The AFL data also show an effect of field position on the number of behinds scored. Not surprisingly, players were less likely to score a behind when they kicked from the boundary line where the relative angle of the goal posts is acute. There were also fewer behinds scored when players were directly in front. This decrease is most likely related to the fact that the other positions encompass 30° of angle whereas the ‘directly in front’ condition does not. There was a strong interaction between the side on which a behind was scored and the side on which the kick was taken. Figure 4 shows that players were more likely to score a behind on the side nearest to them. This effect may be related to the fact that the near behinds can be 6.0 m closer than far behinds. Note that the greater number of right behinds from the right side, relative to left behinds from the left side cannot be attributed to players simply making more attempts from the right side. To our knowledge there is no asymmetry in the game where players attack the goal from the right side of the field. In addition, if this were the case, the left behinds from the right side would be elevated compared to the number of right behinds from the left side. As can be seen in figure 4, the cross field behinds mirror each other in terms of frequency. As such, we conclude that the higher prevalence of right sided misses by AFL players is due to hemispheric asymmetries rather than to any predisposition of players to attempt more kicks from the right side of the field.

In conclusion, the laboratory data show that, when people aim towards the middle of two goal posts placed in far space, they deviate towards the right. This asymmetry in kicking builds on a body of research showing that attentional asymmetries affect a number of everyday activities, such as walking [21], [22], [23] and manoeuvring a wheelchair or electric scooter [24]. The data collected from the field also shows a rightward deviation for goal kicking in the AFL. It would be interesting to see whether other similar sports such as Rugby and American Football show a similar asymmetry in kicking.
